# Transformation of Salicylic Acid and Its Distribution in Tea Plants (*Camellia sinensis*) at the Tissue and Subcellular Levels

**DOI:** 10.3390/plants10020282

**Published:** 2021-02-02

**Authors:** Jianlong Li, Yangyang Xiao, Qian Fan, Yinyin Liao, Xuewen Wang, Xiumin Fu, Dachuan Gu, Yiyong Chen, Bo Zhou, Jinchi Tang, Lanting Zeng

**Affiliations:** 1 Tea Research Institute, Guangdong Academy of Agricultural Sciences & Guangdong Provincial Key Laboratory of Tea Plant Resources Innovation and Utilization, No. 6 Dafeng Road, Tianhe District, Guangzhou 510640, China; skylong.41@163.com (J.L.); chenyiyong@gdaas.cn (Y.C.); zhoubo@gdaas.cn (B.Z.); 2 Guangdong Provincial Key Laboratory of Applied Botany & Key Laboratory of South China Agricultural Plant Molecular Analysis and Genetic Improvement, South China Botanical Garden, Chinese Academy of Sciences, No. 723 Xingke Road, Tianhe District, Guangzhou 510650, China; xiaoyangyang17@scbg.ac.cn (Y.X.); Fanqian@scbg.ac.cn (Q.F.); honey_yyliao@scbg.ac.cn (Y.L.); wangxuewen@scbg.ac.cn (X.W.); fuxiumin@scbg.ac.cn (X.F.); gudachuan@scbg.ac.cn (D.G.); 3 College of Life Sciences, University of Chinese Academy of Sciences, No. 19A Yuquan Road, Beijing 100049, China; 4 National Navel Orange Engineering Research Center, College of Life Sciences, Gannan Normal University, Rongjiang New District, Ganzhou 341000, China; 5 Center of Economic Botany, Core Botanical Gardens, Chinese Academy of Sciences, No. 723 Xingke Road, Tianhe District, Guangzhou 510650, China

**Keywords:** tea, *Camellia sinensis*, salicylic acid, salicylic acid 2-*O*-β-glucoside, methyl salicylate, transformation, distribution

## Abstract

Salicylic acid (SA) is a well-known immune-related hormone that has been well studied in model plants. However, less attention has been paid to the presence of SA and its derivatives in economic plants, such as tea plants (*Camellia sinensis*). This study showed that tea plants were rich in SA and responded differently to different pathogens. Feeding experiments in tea tissues further confirmed the transformation of SA into salicylic acid 2-*O*-β-glucoside (SAG) and methyl salicylate. Nonaqueous fractionation techniques confirmed that SA and SAG were mostly distributed in the cytosol of tea leaves, consistent with distributions in other plant species. Furthermore, the stem epidermis contained more SA than the stem core both in *C. sinensis* cv. “Jinxuan” (small-leaf species) and “Yinghong No. 9” (large-leaf species). Compared with cv. “Yinghong No. 9”, cv. “Jinxuan” contained more SAG in the stem epidermis, which might explain its lower incidence rate of wilt disease. This information will improve understanding of SA occurrence in tea plants and provide a basis for investigating the relationship between SA and disease resistance in tea plants.

## 1. Introduction

Plant hormones are small organic compounds that exist naturally and have critical roles not only in the plant development process, but also in defense and immune responses [1]. Among these important hormones in plants, salicylic acid (SA) participates in many plant growth and development processes, and plays an important role in a plant’s response to environmental stress. Research on SA has mainly focused on plant immunity, as SA is an important immune-related hormone [2]. SA is critical for systemic acquired resistance (SAR), with its accumulation or signal transduction-deficient mutants being unable to produce normal SAR [3,4]. SA can be involved in regulating plant seed germination, flowering, fruit setting, and fruit ripening. Furthermore, SA can mediate plant resistance to abiotic stresses, such as altering plant adaptation to heavy metals, heat, cold, drought, and high-salt stress environments [5]. SA regulation for plant growth and development, and environmental stress response, is achieved by changing the SA concentration and expression intensity of the SA downstream gene. For example, during pathogen infection, SA biosynthesis and signal transduction are enhanced in plants, with SA inducing the expression of disease-resistance-related genes to enhance plant disease resistance. Therefore, understanding the SA biosynthesis and signal transduction processes is an important prerequisite for exploring the plant development and interactions with the environment [2,5,6]. To date, SA has been well studied in model plants [2,5], but comparatively less attention has been directed to certain economic or horticultural plants, such as tea plants (*Camellia sinensis*).

Most tea plants grow in warm and humid tropical and subtropical areas, which are suitable for the propagation of various pathogens. Various types of tea tree pathogens have been identified, with over 100 pathogen-induced tea tree diseases identified in China [7]. However, compared with other horticultural crops, tea tree diseases are not serious, with few reports of tea tree disease causing significant economic loss [8]. This might be due to the evolved defense system in tea plants, which has developed physiological adaptability, resulting in a strong ability to resist infection by pathogens. As a non-model plant, the presence of SA in tea plants has received little attention. To further explore the distribution and transformation of SA and its stress response, this study analyzed changes in the SA content in different tea cultivars (cv. “Jinxuan” and “Yinghong No. 9”) in different months. The transformation of SA in tea plants was analyzed using the stable isotope method, and the distributions of SA and its transformation products were analyzed at the tissue and cellular levels. This information will advance our understanding of the occurrence of SA in tea plants and provide a basis for investigating the relationship between SA and disease resistance in tea plants.

## 2. Results and Discussion

### 2.1. Occurrence of SA in Tea Leaves and Its Response to Different Pathogens

**Figure 1:**
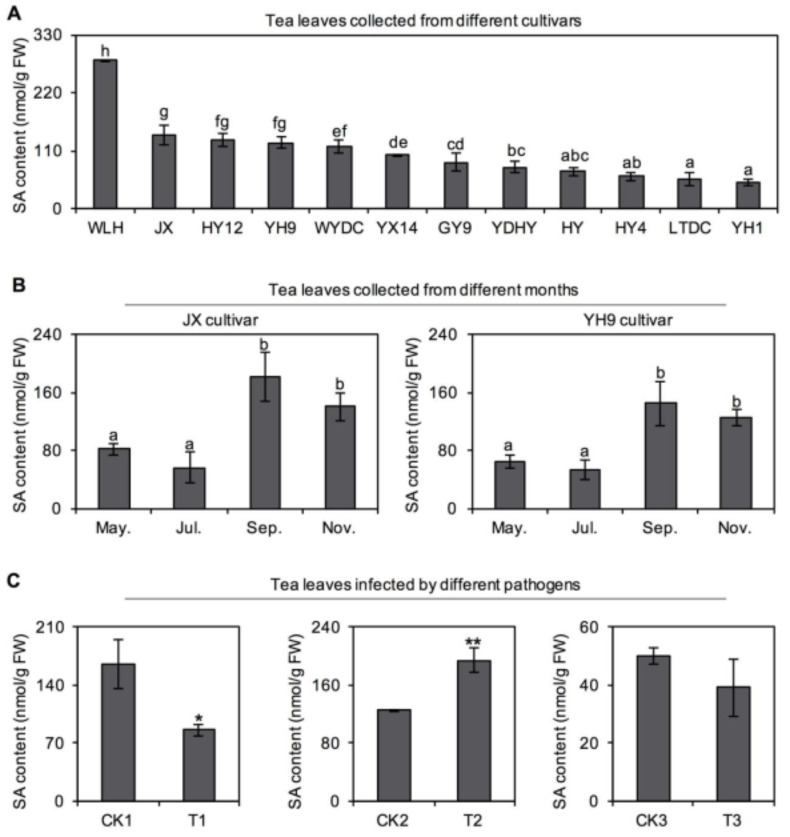
Occurrence of salicylic acid (SA) in normal and pathogen-infected tea leaves. (**A**) Differential occurrence of SA in normal tea leaves from different cultivars. The samples were collected in November 2018 and from 12 tea cultivars including WLH (Wulinghong), JX (Jinxuan), HY12 (Hongyan No. 12), YH9 (Yinghong No. 9), WYDC (Wuyedancong), YX14 (Youxuan No. 14), GY9 (Guanyin No. 9), YDHY (Yundaheiye), HY (Huangyu), HY4 (Hongyan No. 4), LTDC (Lingtoudancong), and YH1 (Yinghong No. 1). (**B**) Differential occurrence of SA in normal tea leaves from different plucked months. May., Jul., Sep., and Nov. represent the tea leaves plucked in May 2020, July 2019, September 2019, and November 2018, respectively. Means with different letters are significantly different from each other (*p* ≤ 0.05). (**C**) Change in content of SA in tea leaves after pathogen infection. The 3rd and 4th leaves infected by *Cephaleuros virescens* Kunge and collected in Jul 2020 were used as T1, and ones without infection were used as CK1. The old leaves infected by *Colletotrichum camelliae* collected in Jul 2020 were used as T2, and ones without infection were used as CK2. The buds infected by *Fusarium* collected in Jun 2020 were used as T3, and ones without infection were used as CK3. All samples were collected from tea cultivar (Yinghong No. 9). Significant differences are indicated as * (*p* ≤ 0.05) and ** (*p* ≤ 0.01). All data are expressed as mean ± SD (*n* = 3). FW, fresh weight.

In China, cultivated tea plants are mainly classified into two groups, namely, large-leaf and small-leaf species [9]. In this study, 12 different tea cultivars, comprising six large-leaf species and six small-leaf species, were selected to investigate SA occurrence in normal leaves. The investigation of these different tea cultivars showed that SA was highly distributed in tea leaves, ranging from 50 to 300 nmol/g of fresh weight (Figure 1A). Two cultivars widely planted in South China, *C. sinensis* cv. “Jinxuan” and “Yinghong No. 9”, were used to further explore the SA distribution in tea leaves in different seasons. High SA accumulation in tea leaves was found to be dependent on the plucking month (Figure 1B). The SA amounts in tea leaves from *C. sinensis* cv. “Jinxuan” and “Yinghong No. 9” plucked in May and July were lower than in those plucked in September and November. The SA content response in tea plants after pathogen infection was also studied. Changes in SA content in tea leaves after infection by three different pathogens, namely, *Cephaleuros virescens* Kunze, *Colletotrichum camelliae*, and *Fusarium* showed that high levels of accumulated SA responded differently to different pathogens (Figure 1C).

SA is a signal substance, which has important roles in plant responses to various stresses and environmental adaptations [5]. SA mainly functions by changing concentration and eliciting defense mechanisms downstream. Therefore, many plants generally contain low SA levels when not stimulated by stress. For example, *Cucumis sativus*, *Nicotiana tabacum*, *Arabidopsis thaliana*, and *Capsicum annuum*, generally have SA concentrations of less than 1 nmol/g of fresh weight without stress [10]. The amount of SA is significantly increased in these plants when infected by pathogens, ranging from 1–150 nmol/g of fresh weight after stress [10]. However, in this study, the SA concentration in tea plants was above 50 nmol/g of fresh weight without stress (Figure 1), consistent with previous studies [8,11,12]. An order of magnitude difference in the SA content was observed between tea plants and other species. Furthermore, this high SA accumulation was unchanged among tea cultivars and plucking seasons. Therefore, potential factors responsible for the relatively high concentration of SA in tea plants, such as increased amounts of the SA synthesis precursor, the high catalytic activity of key synthases, or induction by endophytes unique to tea plants, were of interest. Relevant research requires in-depth development in the future. In general, plants with low SA contents show significantly increased content after pathogen attack. However, what happens to the high SA content in plants after infection is also of interest. As one of only a few reported species with high SA accumulation, rice (*Oryza sativa*) shows no significant change in SA content after infection by pathogens [13]. However, in tea plants, SA showed different changes after infection by different pathogens (Figure 1C). Therefore, in addition to acting as a constitutive defense mechanism, SA might also be an inducible defense mechanism in tea plants.

### 2.2. Transformation of SA into SAG and Methyl Salicylate (MeSA) in Tea Tissues

**Figure 2:**
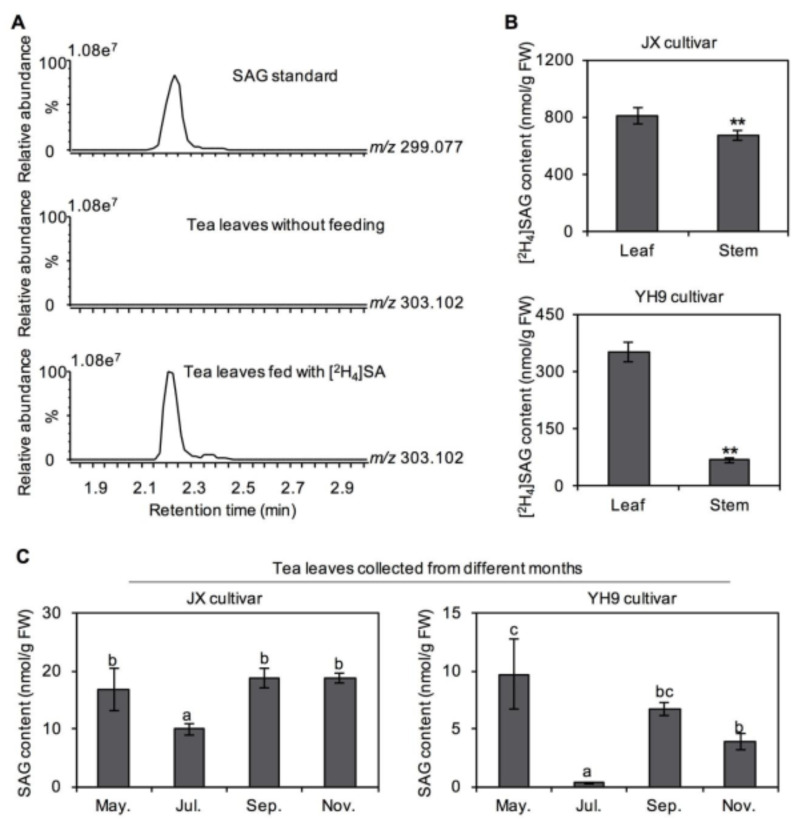
Transformation of salicylic acid (SA) into salicylic acid 2-*O*-β-glucoside (SAG) in tea plants. (**A**) Identification of [^2^H_4_]SAG in tea leaves fed with [^2^H_4_]SA. (**B**) Differential transformation ability of SA into SAG in different tea cultivars and tissues. *Camellia sinensis* cv. Jinxuan (JX) and Yinghong No. 9 (YH9) were used for investigation. Data are expressed as mean ± SD (*n* = 4). Significant differences are indicated as * (*p* ≤ 0.05) and ** (*p* ≤ 0.01). (**C**) Differential occurrence of SAG in normal tea leaves plucked in different months. May., Jul., Sep., and Nov. represent the tea leaves plucked in May 2020, July 2019, September 2019, and November 2018, respectively. Data are expressed as mean ± SD (*n* = 3). Means with different letters are significantly different from each other (*p* ≤ 0.05). FW, fresh weight.

**Figure 3:**
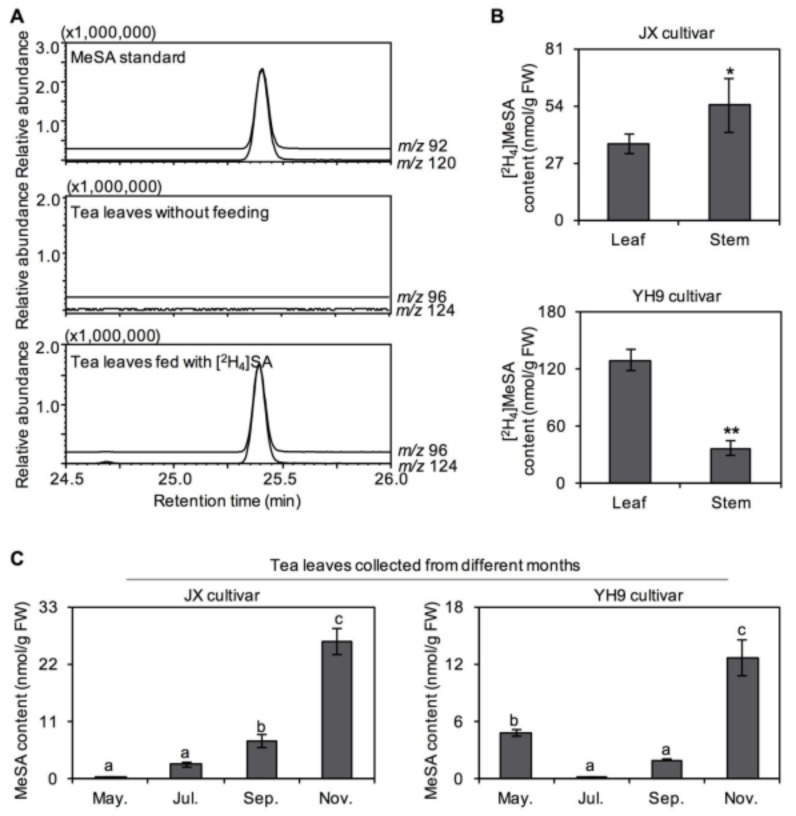
Transformation of salicylic acid (SA) into methyl salicylate (MeSA) in tea plants. (**A**) Identification of [^2^H_4_]MeSA in tea leaves fed with [^2^H_4_]SA. (**B**) Differential transformation ability of SA into MeSA in different tea cultivars and tissues. *Camellia sinensis* cv. Jinxuan (JX) and Yinghong No. 9 (YH9) were used for investigation. Data are expressed as mean ± SD (*n* = 4). Significant differences are indicated as * (*p* ≤ 0.05) and ** (*p* ≤ 0.01). (**C**) Differential occurrence of MeSA in tea leaves plucked in different months. May., Jul., Sep., and Nov. represent the tea leaves plucked in May 2020, July 2019, September 2019, and November 2018, respectively. Data are expressed as mean ± SD (*n* = 3). Means with different letters are significantly different from each other (*p* ≤ 0.05). FW, fresh weight.

In plants, in addition to acting as a hormone signaling substance, SA can be a precursor substance for conversion into some important metabolites. In this study, in vivo evidence of SA transformation in tea plants was obtained by a stable isotope feeding experiment. The fourth leaf combined with the stem from *C. sinensis* cv. “Jinxuan” and “Yinghong No. 9” was fed with [^2^H_4_]SA and subjected to metabolite analysis. After the feeding experiment, the leaf and stem were collected separately and the contents of labelled derivatives were determined. [^2^H_4_]SAG and [^2^H_4_]MeSA were identified by ultra-performance liquid chromatography–quadrupole time-of-flight mass spectrometry (UPLC–QTOF–MS) and gas chromatography–mass spectrometry (GC–MS), respectively (Figure 2A and Figure 3A). Quantitative analysis of the labeled derivatives showed that the transformation of SA into SAG was greater in *C. sinensis* cv. “Jinxuan” than in *C. sinensis* cv. “Yinghong No. 9” (Figure 2B), with the opposite result obtained for MeSA (Figure 3B). The SAG and MeSA contents in tea leaves plucked in different months were also monitored. The absolute amounts of SAG and MeSA in tea leaves were lower than that of SA in both *C. sinensis* cv. “Jinxuan” and “Yinghong No. 9” (Figure 2C and Figure 3C). The amounts of MeSA in tea leaves from *C. sinensis* cv. “Jinxuan” and “Yinghong No. 9” slightly increased from May to November (Figure 3C), which was consistent with changes in the SA content.

The isotope tracing technique is a classical and effective method, especially in plants lacking a genetic transformation system, such as tea plants. In vivo tracking using a stable isotope-labeled precursor allows for the secondary metabolic pathways to be identified or confirmed. Tea plants are a species, among others, that contain abundant secondary metabolites [8,10,14]. Many studies have used this method to study the biosynthesis of secondary metabolites in tea plants [15,16,17]. In the present study, SA was found to be metabolized into SAG and MeSA in tea tissues using stable isotope tracing (Figure 2 and Figure 3), consistent with SA metabolism in other species [18]. After synthesis, many chemical modifications of SA can occur, such as glucosylation and methylation. Most modifications result in SA being transformed into its inactive state, while maintaining its level of accumulation, function, and/or mobility within finely tuned parameters. For example, glucosylation inactivates SA, and glycosylated substances were stored in the vacuolar at relatively large quantities. Glucose conjugation at the hydroxyl group of SA leads to the biosynthesis of SAG as a major conjugate [18]. Methylation of SA can enhance its membrane permeability and volatility. For example, an SA carboxyl methyltransferase can also convert SA into MeSA, a volatile derivative [19,20].

### 2.3. Distribution of SA and Its Derivatives in Tea Plants at Tissue and Subcellular Levels

**Figure 4:**
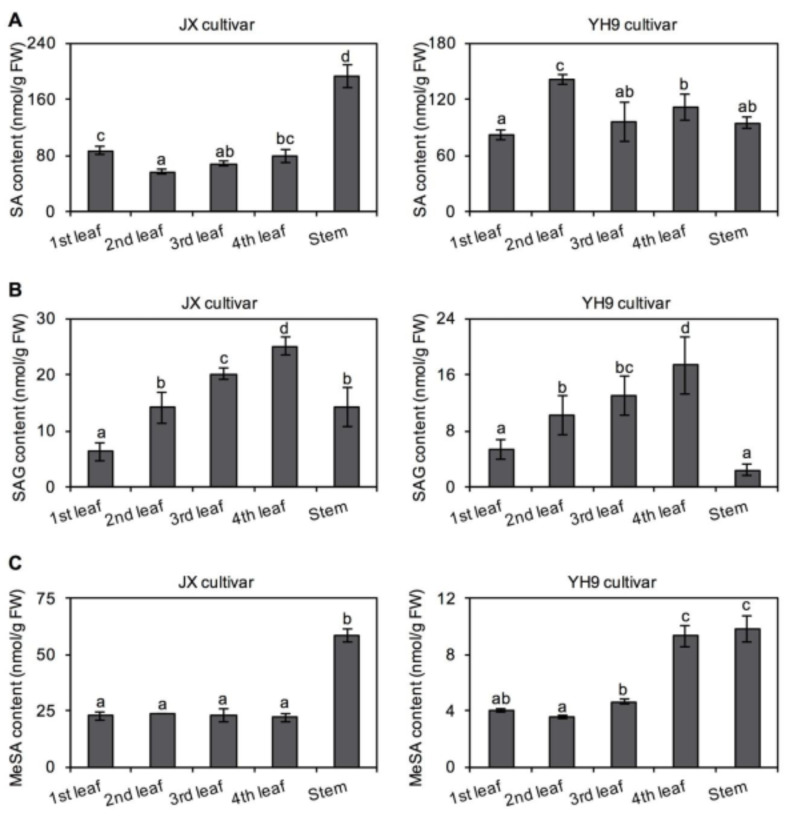
Differential occurrence of salicylic acid (SA) (**A**), salicylic acid 2-*O*-β-glucoside (SAG) (**B**) and methyl salicylate (MeSA) (**C**) in tea leaves with different positions and stem. *Camellia sinensis* cv. Jinxuan (JX) and Yinghong No. 9 (YH9) collected in May 2020 were used for investigation. Data are expressed as mean ± SD (*n* = 3). Means with different letters are significantly different from each other (*p* ≤ 0.05). FW, fresh weight.

**Figure 5:**
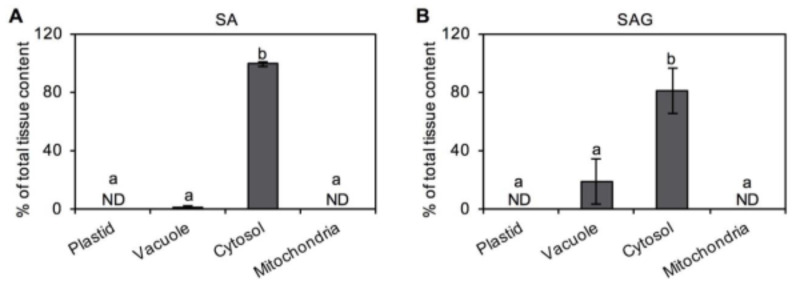
Subcellular distributions of (**A**) salicylic acid (SA) and (**B**) its derivative salicylic acid 2-*O*-β-glucoside (SAG) in tea leaves. Tea leaves were from *Camellia sinensis* cv. Jinxuan plant. The tissues of tea leaf were fractionated using a nonaqueous procedure. SA and SAG in each fraction were measured by ultrahigh-performance liquid chromatography. The subcellular distributions were calculated by comparing the metabolite and marker enzyme distributions using Bestfit software. Data expressed as mean ± standard deviation (SD) (*n* = 3). Means with different letters are significantly different from each other (*p* ≤ 0.05). ND, not detected.

In addition to different plucking months, the contents of SA, SAG, and MeSA were determined in tea leaves from different leaf positions and stems. In *C. sinensis* cv. “Jinxuan” samples, the contents of these metabolites showed a significant trend (Figure 4). However, in *C. sinensis* cv. “Yinghong No. 9”, no conclusion could be drawn regarding changes in the contents of these metabolites. Furthermore, the distribution of SA and its derivatives in tea leaves at the subcellular level was investigated. SA and SAG both showed the highest accumulation in the cytosol of tea leaves, with a minor amount of SAG distributed in vacuoles (Figure 5).

The lack of a genetic transformation system results in difficulties in obtaining in vivo evidence of the locations of metabolite biosynthesis in tea plants. Direct investigation of the metabolite localization at the cellular and subcellular levels might help solve this problem. The nonaqueous fractionation (NAF) method has been widely used to investigate the distribution of metabolites in many plant species [21,22]. Recently, NAF has been successfully applied in determining the characteristic metabolites from tea plants [15,23]. In the present study, the subcellular distributions of SA and SAG was revealed by the NAF method. Their distributions in tea plants were consistent with those in other plant species. SA is synthesized by plants through two pathways, namely, the isochorismate synthase (ICS) and phenylalanine ammonia-lyase (PAL) pathways [24]. These pathways both begin in chloroplasts with the chorismate precursor and involve multiple enzymatic reactions. The contributions of these two pathways to SA synthesis varies. In *Arabidopsis thaliana*, immune-associated SA is mainly produced by the ICS pathway. Recently, important advances have been made in terms of SA biosynthesis. The downstream pathway of isochorismate needs two proteins, ENHANCED DISEASE SUSCEPTIBILITY5 (exporting isochorismate from the plastid to the cytosol) and cytosolic amidotransferase avrPphB SUSCEPTIBLE3 (PBS3) [25]. Subcellular localization analysis showed that PBS3 was localized in the cytosol [25]. Furthermore, SAG is actively transported from the cytosol to the vacuole, where it can act as an inactive storage form of free SA for release [26]. Therefore, some SAG was also found in vacuoles (Figure 5).

### 2.4. Spatial Distribution of SA and Its Derivatives in Tea Stem

**Figure 6:**
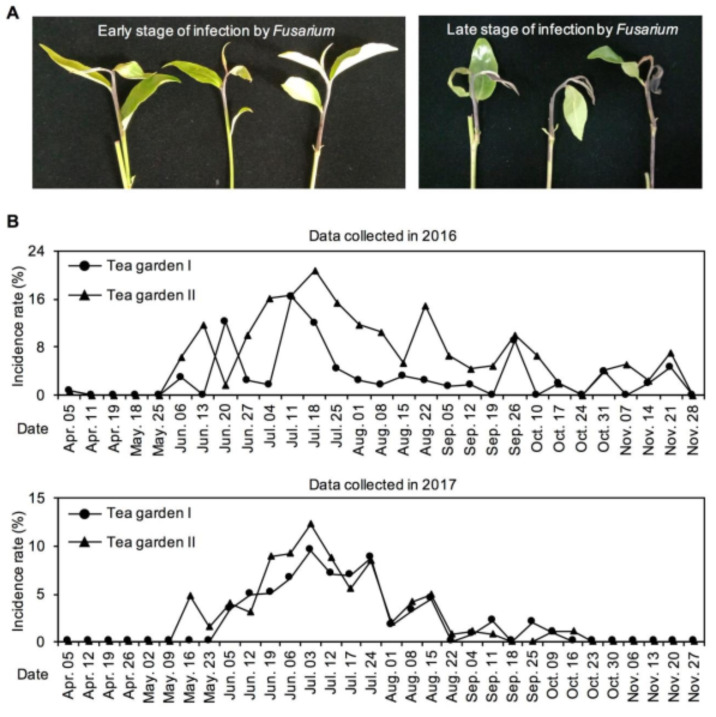
(**A**) Phenotypes of tea branches after natural infection by *Fusarium*. The branches from *Camellia sinensis* cv. “Yinghong No. 9” was recorded. (**B**) Incidence rate of wilt disease in *C. sinensis* cv. “Yinghong No. 9” plant. The data were collected from two tea gardens. Data are expressed as mean (*n* = 3).

**Figure 7:**
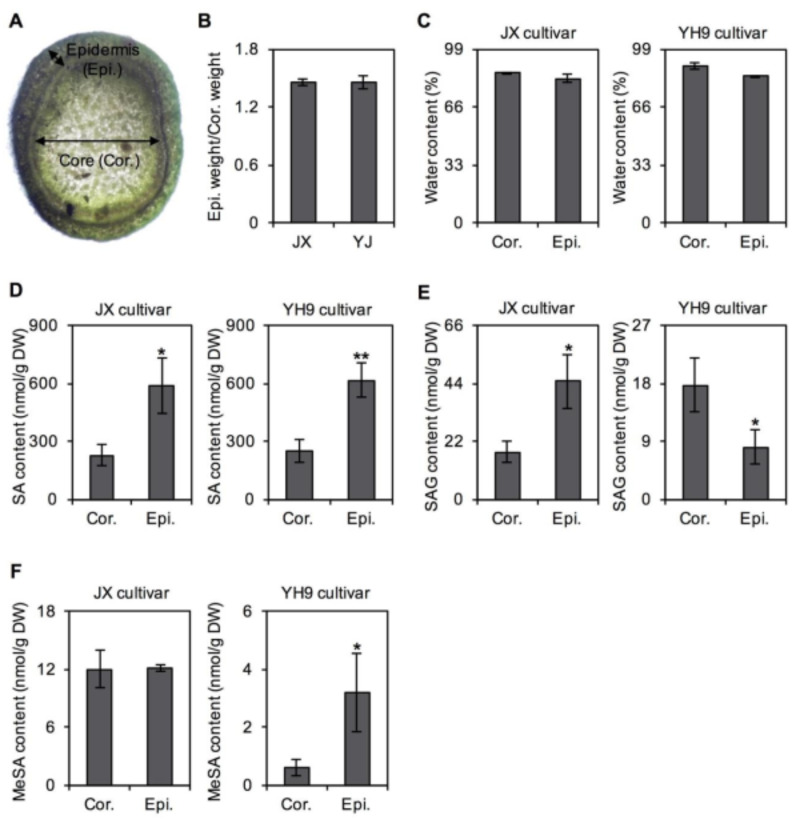
Occurrence of salicylic acid (SA) and its transformation in different parts of tea stem. (**A**) Cross section of tea stem. (**B**) The ratio of epidermis weight to core weight. (**C**) The water content of epidermis and core of tea stem. (**D**) Differential occurrence of SA in epidermis and core of tea stem. (**E**) Differential occurrence of salicylic acid 2-*O*-β-glucoside (SAG) in epidermis and core of tea stem. (**F**) Differential occurrence of methyl salicylate (MeSA) in epidermis and core of tea stem. *Camellia sinensis* cv. Jinxuan (JX) and Yinghong No. 9 (YH9) collected in September 2020 were used for investigation. Data are expressed as mean ± SD (*n* = 3). Significant differences are indicated as * (*p* ≤ 0.05) and ** (*p* ≤ 0.01).

*C. sinensis* cv. “Yinghong No. 9” is widely planted in South China, especially Guangdong Province, and is suitable for processing into a black tea, which is very popular. Recently, wilt disease has been noted as one of the most serious diseases in *C. sinensis* cv. “Yinghong No. 9” plants. This causes the stem of infected branches to wither, contract, and bend, and the shoot to stop growing and eventually turn black, leading to reductions in tea quality and yield (Figure 6A). Usually, the disease begins in May, became more serious in June, before reaching its peak in July and August, and then declined in early October. In this study, the statistics for two tea gardens from 2016 to 2017 showed that wilt disease mainly occurred in *C. sinensis* cv. “Yinghong No. 9” plants between June and August, and the incidence rate ranged from 2–16% (Figure 6B). However, another tea cultivar widely planted in South China, *C. sinensis* cv. “Jinxuan”, essentially showed no outbreak of wilt disease, and the incidence rate was less than 1% between June and August. Since very few plants (cv. “Jinxuan”) were infected, the incidence data are not shown in Figure 6B. A significant difference was observed in the incidence rate of wilt disease between the two cultivars. Tea wilt disease is a fungal disease, with infection beginning primarily at the stem site. Therefore, in this study, the spatial distribution of SA and its derivatives in tea stems was also investigated. The results showed that SA accumulated in the epidermis of the two cultivars, while SAG showed the opposite distribution (Figure 7).

Presently, only a few studies on tea wilt disease have been published. Liu et al. firstly discovered the disease and studied its physiological characteristics [27]. According to the morphological characteristics of conidia, the pathogen responsible for wilt disease is a species of *Diplodia frumenti* [28]. Further isolation and purification of the pathogen indicated that it is a *Fusarium* species. However, as the gene sequence of this pathogen has not been reported, genetic information of tea wilt disease requires further study. According to the research and tea garden observations, this disease mainly harmed large-leaf species [27]. In the present study, *C. sinensis* cv. “Yinghong No. 9” was a large-leaf species, while *C. sinensis* cv. “Jinxuan” was a small-leaf species. Higher SAG accumulation was observed in the stem epidermis of *C. sinensis* cv. “Jinxuan” compared with that of *C. sinensis* cv. “Yinghong No. 9” (Figure 7), which might result in less wilt disease infection in the former. In plants, large amounts of SA are generally converted into SAG, which is inactive and nontoxic [18]. Under stress, SAG can be hydrolyzed into active SA, activating the defense system [26].

In addition, we have tried our best to establish the pathogen infection system in tea plants. However, at present, only the pathogen can be isolated, being successfully identified as *Fusarium*, responsible for inducing wilt disease in tea plants. However, it has not been successfully inoculated into tea plants, even though many inoculation methods have been tried. Inoculation experiments have been carried out in *C. sinensis* cv. “Yinghong No. 9” in the experimental fields and laboratory. In the experimental field, the normal tea plants were sprayed or injected with a solution of *Fusarium*. After inoculation for a week, the tea buds grew normally and no infection symptoms were found. A high accumulation of defensive substances, such as SA, caffeine, and gallic acid [8], might be responsible for the failure of inoculation. Furthermore, there are many other unknown factors under the field conditions, so the real cause of the failure has yet to be determined. In the laboratory, the branches of *C. sinensis* cv. “Yinghong No. 9” were injected with a spore suspension of *Fusarium*. After injection and cultivation for several days, both control and infected tea leaves were blackened and fell off.

## 3. Materials and Methods

### 3.1. Plant Materials and Treatment

All tea samples were collected from Yingde Tea Experimental Station (Yingde, Guangdong, China) of the Tea Research Institute, Guangdong Academy of Agricultural Sciences. The details of sample preparation or sampling, including cultivar, type, state and collection period, are summarized in Table 1.

Twelve different tea plant cultivars including Wulinghong, Jinxuan, Hongyan No. 12, Yinghong No. 9, Wuyedancong, Youxuan No. 14, Guanyin No. 9, Yundaheiye, Huangyu, Hongyan No. 4, Lingtoudancong, Yinghong No. 1 were collected in November 2018. The leaves from healthy *C. sinensis* cv. “Jinxuan”, and “Yinghong No. 9” plants were sampled in May 2020, July and September 2019. The 3rd and 4th leaves infected by *Cephaleuros virescens* Kunge or without infection were collected from *C. sinensis* cv. “Yinghong No. 9” in Jul 2020. The old leaves infected by *Colletotrichum camelliae* or without infection were collected from *C. sinensis* cv. “Yinghong No. 9” in Jul 2020. The buds infected by *Fusarium* or without infection from *C. sinensis* cv. “Yinghong No. 9” were collected in Jun 2020.

To investigate the transformation ability of SA into MeSA and SAG in different tea cultivars, the 4th tea leaf containing the stem from different cultivars (*C. sinensis* cv. “Jinxuan” and “Yinghong No. 9”) collected in August 2020 was subjected to isotope tracing experiment. The samples were cultivated in 6 mM [^2^H_4_]SA (nearly 500 μL) under continuous light for 14 h. The samples were kept at 70% humidity and 25 °C. After feeding, the tea leaf and stem were collected separately. Four replicates were processed in the study.

To investigate the spatial distribution of SA and its derivatives in the tea stem, the stem between the 2rd and 4th leaves from different cultivars (*C. sinensis* cv. “Jinxuan” and “Yinghong No. 9”) were collected in September 2020. The epidermis and corn of the stem were separated and subjected to metabolite analysis. Three replicates were processed in the study.

### 3.2. Extraction and Analysis of SA

For the extraction and analysis of SA in tea samples we referred to the previous study [11]. Finely powdered sample was extracted with ethyl acetate. After extraction, the extract was subjected to UPLC–QTOF–MS analysis. The details of the extraction and analysis of SA are provided in the Supplementary Materials.

### 3.3. Extraction and Analysis of MeSA and [^2^H_4_]MeSA

For the extraction and analysis of volatile compounds we referred to the previous study [16]. Finely powdered sample was extracted with dichloromethane containing ethyl decanoate as an internal standard. After the extraction, the extract was subjected to gas chromatography–mass spectrometry (GC–MS) analysis. The details about the extraction and analysis of MeSA and [^2^H_4_]MeSA are provided in the Supplementary Materials. The characteristic ions of MeSA and [^2^H_4_]MeSA are m/z 120 and 124.

### 3.4. Extraction and Analysis of SAG and [^2^H_4_]SAG

The sample was extracted with 70% methanol, and the extract was subjected to UPLC–QTOF–MS analysis. The details about the extraction and analysis of SAG and [^2^H_4_]SAG are provided in the Supplementary Materials. The characteristic ion of SAG is *m/z* 299.0770. Because of the high level of similarity to most other peptides in electrospray and matrix-assisted laser desorption ionization, leucine enkephalin is a convenient model compound for the routine testing of instrument conditions [29]. Therefore, the calibration of the peak area was based on the leucine enkephalin.

### 3.5. Determination of Subcellular Distribution of SA and SAG

The tea leaves from *C. sinensis* cv. Jinxuan plucked in March 2019 were fractionated using a nonaqueous procedure, in accordance with previously published studies, with a slight modification [15,22,30,31]. The details regarding the separation are provided in the Supplementary Materials. After the separation, the sample was extracted for assay of the metabolites and enzymes (proteins). The metabolites (SA and SAG) from the dried samples were resolved in ethyl acetate, and subjected to ultrahigh-performance liquid chromatography (UHPLC) analysis. Selected reaction monitoring (SRM) mode was used in MS/MS instrument and the SRM transitions of SA and SAG are listed in Table 2. Enzyme activity analysis of markers were used to determine the amount of organelles. The related details are provided in the Supplementary Materials.

### 3.6. Analysis of Incidence Rate of Wilt Disease in Tea Gardens

The data were collected from two tea gardens, in the Yingde Tea Experimental Station (Yingde, Guangdong, China) of the Tea Research Institute, Guangdong Academy of Agricultural Sciences. Only the incidence rate of wilt disease in C. sinensis cv. “Yinghong No.9” plants was analyzed from 2016 to 2017. During the investigation, three investigation sites (0.33 m^2^) were selected randomly, and the number of new shoots and infected shoots in each site was recorded.

### 3.7. Statistical Analysis

SPSS Statistics package (Version 23.0) was applied to carry out the statistical analysis. The differences between two groups and among three, or more than three, groups were determined by two-tailed Student’s t test and one-way analysis of variance (ANOVA) followed by Duncan’s multiple comparison tests, respectively. A probability level of 5% (*p* ≤ 0.05) was regarded as significant. All data are expressed as the mean ± standard deviation (SD).

## 4. Conclusions

**Figure 8:**
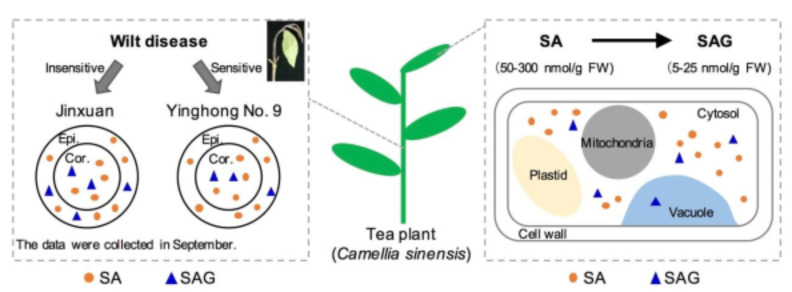
Summary of the high accumulation and distribution of salicylic acid (SA) and salicylic acid 2-*O*-β-glucoside (SAG) in tea plant. Epi., epidermis; Cor., core.

In this study, multidomain methods were applied to comprehensively study the occurrence and transformation of SA in tea plants (Figure 8). The abundant accumulation of SA in tea leaves was independent of the cultivar and plucking month, with different responses observed in response to different pathogens. Isotope feeding experiments confirmed that, as in other plant species, SA was transformed into SAG and MeSA in tea plants. In vivo evidence of the distribution of SA and SAG in tea leaves was obtained by NAF, with these metabolites mostly being located in the cytosol of tea leaves. Furthermore, the epidermis and core of the stem from two different cultivars were separated and subjected to content analysis of SA and its derivatives. The spatial distribution results showed that differential accumulation of SAG in the stem epidermis between *C. sinensis* cv. “Jinxuan” and “Yinghong No. 9” might be responsible for their different incidence rates of wilt disease. This study will aid the understanding of the distribution of SA and its resistant derivatives in tea plants and provide a basis for investigating why tea plants have few diseases.

## Figures and Tables

**Table 1:** The details of sample preparation or sampling in the study.

Cultivar	Type	State	Collection Period
Wulinghong	One bud and three leaves	Healthy	November 2018
Jinxuan			
Hongyan No. 12			
Yinghong No. 9			
Wuyedancong			
Youxuan No. 14			
Guanyin No. 9			
Yundaheiye			
Huangyu			
Hongyan No. 4			
Lingtoudancong			
Yinghong No. 1			
Jinxuan	One bud and three leaves, leaves with different positions, stem	Healthy	May 2020
Yinghong No. 9			
Jinxuan	One bud and three leaves	Healthy	July and September 2019
Yinghong No. 9			
Jinxuan	Fourth leaf combined with stem	Healthy	August 2020
Yinghong No. 9			
Jinxuan	Epidermis and corn of stem	Healthy	September 2020
Yinghong No. 9			
Yinghong No. 9	Third and fourth leaves	Healthy and infected by *Cephaleuros virescens* Kunge	July 2020
Yinghong No. 9	Old leaf	Healthy and infected by *Colletotrichum camelliae*	July 2020
Yinghong No. 9	Bud	Healthy and infected by *Fusarium*	June 2020

**Table 2:** Precursor and products ions of authentic standards used in the UHPLC−MS/MS analysis.

Compound	Retention Time (Min)	Transitions (*m/z*)	Collision Energy (V)
SA	9.20	137.06→65.33	28.15
		137.06→75.22	30.58
		137.06→93.17	15.56
SAG	5.51	299.00→93.15	33.16
		299.00→137.06	10.25
		299.00→161.00	10.25

SA, salicylic acid; SAG, salicylic acid 2-*O*-β-glucoside; UHPLC−MS/MS, ultrahigh-performance liquid chromatography−tandem mass spectrometry.

## Data Availability

The data presented in this study are openly available in FigShare at https://doi.org/10.3390/xxxxx.

## Supplementary Materials

The following are available online at https://www.mdpi.com/2223-7747/10/2/282/s1.

## Abbreviations

FW	Fresh weight
GC–MS	Gas chromatography–mass spectrometry
MeSA	Methyl salicylate
NAF	Nonaqueous fractionation
SA	Salicylic acid
SAG	Salicylic acid 2-*O*-β-glucoside
UHPLC	Ultrahigh-performance liquid chromatography
UPLC–QTOF–MS	Ultra−performance liquid chromatography/quadrupole time−of−flight mass spectrometry
