# Supplementary material for: Transformation of Salicylic Acid and Its Distribution in Tea Plants (Camellia sinensis) at the Tissue and Subcellular Levels
Source: Plants (Basel). 2021 Feb 2;10(2):282. doi: 10.3390/plants10020282 (PMC7912924; doi:10.3390/plants10020282)
Supplement: Supplementary file 1 [file plants-10-00282-s001.pdf]

plants

SUPPLEMENTARY INFORMATION

Transformation of salicylic acid and its distribution in tea plants (*Camellia sinensis*) at the tissue and subcellular levels

Jianlong Li [1,†], Yangyang Xiao [2,3,†], Qian Fan [2,4], Yinyin Liao [2,3], Xuewen Wang [2,3], Xiumin Fu [2], Dachuan Gu [2], Yiyong Chen [1], Bo Zhou [1], Jinchi Tang [1,*] and Lanting Zeng [2,5,*]

[1] Tea Research Institute, Guangdong Academy of Agricultural Sciences & Guangdong Provincial Key Laboratory of Tea Plant Resources Innovation and Utilization, No. 6 Dafeng Road, Tianhe District, Guangzhou 510640, China; skylong.41@163.com (J.L.); chenyiyong@gdaas.cn (Y.C.); zhoubo@gdaas.cn (B.Z.)
[2] Guangdong Provincial Key Laboratory of Applied Botany & Key Laboratory of South China Agricultural Plant Molecular Analysis and Genetic Improvement, South China Botanical Garden, Chinese Academy of Sciences, No. 723 Xingke Road, Tianhe District, Guangzhou 510650, China; xiaoyangyang17@scbg.ac.cn (Y.X.); Fanqian@scbg.ac.cn (Q.F.); honey_yyliao@scbg.ac.cn (Y.L.); wangxuewen@scbg.ac.cn (X.W.); fuxiumin@scbg.ac.cn (X.F.); gudachuan@scbg.ac.cn (D.G.)
[3] College of Life Sciences, University of Chinese Academy of Sciences, No.19A Yuquan Road, Beijing 100049, China
[4] National Navel Orange Engineering Research Center, College of Life Sciences, Gannan Normal University, Rongjiang New District, Ganzhou 341000, China
[5] Center of Economic Botany, Core Botanical Gardens, Chinese Academy of Sciences, No. 723 Xingke Road, Tianhe District, Guangzhou 510650, China
* Correspondence: tangjinchi@126.com, Tel.: +86-20-8516-1049; zenglanting@scbg.ac.cn, Tel.: +86-20-3702-1938
† These authors contributed equally to this work.

Citation: Li, J.; Xiao, Y.; Fan, Q.; Liao, Y.; Wang, X.; Fu, X.; Gu, D.; Chen, Y.; Zhou, B.; Tang, J.; et al. Transformation of salicylic acid and its distribution in tea plants (*Camellia sinensis*) at the tissue and subcellular levels. **2021**, *9*, x. https://doi.org/10.3390/xxxxx

Received: 06 December 2020
Accepted: 14 December 2021
Published: date

Publisher's Note: MDPI stays neutral with regard to jurisdictional claims in published maps and institutional affiliations.

Copyright: © 2021 by the authors. Submitted for possible open access publication under the terms and conditions of the Creative Commons Attribution (CC BY) license (http://creativecommons.org/licenses/by/4.0/).

Materials and Methods

1.. Extraction and Analysis of Salicylic Acid (SA)

The extraction and analysis of SA in tea samples were referred to the previous study [1]. Finely powdered sample (300 mg, fresh weight) was extracted with 3 mL ethyl acetate by vortexing for 30 s followed by ultrasonic extraction in ice-cold water for 20 min. After centrifuging at 10000×g for 5 min at 4 °C, 2.5 mL supernatants were collected. [$^2H_4$]SA as an internal standard was added to supernatants before dried under a stream of nitrogen. The residue was re-dissolved in 200 μL methanol. The supernatants were filtered through a 0.22 μm membrane, and subjected to an ultra–performance liquid chromatography–quadrupole time–of–flight mass spectrometry (UPLC–QTOF–MS) (Acquity UPLC I-Class/Xevo® G2-XS QTOF, Waters Corporation, MA, USA). Each sample (2 μL) was injected onto a Waters ACQUITY UPLC HSS T3 C18 column (2.1 mm×100 mm, 1.8 μm). Solvent A was Milli-Q water with 0.1% (v/v) formic acid. Solvent B was acetonitrile with 0.1% (v/v) formic acid. The solvent gradient was started at 20% B, then linearly increased to 35% within 10 min, later increased to 95% B in 0.1 min and kept for 3 min. In that moment, it suddenly dropped to 20% in 0.1 min and maintain for 3 min. The flow rate was 0.4 mL/min. The column temperature was 30 °C. The electrospray ionization operated on negative mode. The MS conditions were capillary voltage: 1.5 kV; source temperature: 100 °C; desolvation temperature: 300 °C; cone gas flow: 50 L/ h; and desolvation gas flow: 600 L/ h. The quantitative analysis of SA in tea samples was based on the authentic standard.

2. Extraction and Analysis of Methyl Salicylate (MeSA) and [$^2H_4$]MeSA

Extraction and analysis of volatile compounds were referred to the previous study [2]. Finely powdered sample (200 mg) was extracted with dichloromethane (1.8 mL) containing ethyl decanoate (0.5 nmol) as an internal standard using a shaker at room temperature for 5–6 h. The extraction solution was collected, dried using anhydrous sodium sulfate, and concentrated to 50–100 μL under a stream of nitrogen. The extract (1 μL) was then subjected to gas chromatography–mass spectrometry (GC–MS) analysis carried on a

GC–MS QP2010 SE (Shimadzu Corporation, Kyoto, Japan) equipped with GCMS Solution software (Version 2.72, Shimadzu Corporation, Japan). Samples were injected into the GC injection port held at 230 °C for 1 min, with all injections made in splitless mode. Volatile compounds were separated on a SUPELCOWAX 10 column (30 m × 0.25 mm × 0.25 μm, Supelco Inc., Bellefonte, PA, USA). Helium was used as the carrier gas, with a velocity of 1 mL/min. The initial GC oven temperature was 60 °C for 3 min, which was ramped up to 240 °C at a rate of 4 °C/min, and then held at 240 °C for 20 min. Mass spectrometry was operated in full scan mode (mass range, m/z 40–200). The characteristic ions of MeSA and $[^2H_4]$MeSA are m/z 120 and 124, respectively. The quantitative analyses of MeSA and $[^2H_4]$MeSA in tea samples were based on the unlabeled MeSA standard.

3. Extraction and Analysis of Salicylic Acid 2-O-β-Glucoside (SAG) and $[^2H_4]$SAG

Sample was extracted with 1 mL 70% methanol and vortexed for 30 s. The mixture followed by ultrasonic extraction in ice-cold water for 10 min. After centrifuging at 10000 *g* for 10 min at 4 °C, supernatants were collected and filtered through a 0.22 μm membrane, then subjected to an UPLC–QTOF–MS (Acquity UPLC I-Class/Xevo® G2-XS QTOF, Waters Corporation, MA, USA) equipped with LCMS Solution software (Masslynx v 4.1, Waters Corporation, MA, USA). Sample (2 μL) was injected and separated by a ACQUITY UPLC-BEH C18 column (100 mm×2.1 mm,1.7 μm; Waters Corporation). A binary mobile phase of Milli-Q water with 0.1% (v/v) formic acid (A) and acetonitrile with 0.1% (v/v) formic acid (B) was used. The flow rate was set as 0.3 mL/min. The gradient was set as follows: 0 min (10% B), 5 min (50% B), 5.1 min (90% B), 8 min (90% B), 8.1 min (10% B), and 12 min (10% B). The column oven temperature was 35 °C. The electrospray ionization (ESI) was operated in the negative ion mode, spray voltasge set at 2000V, source temperature was 100 °C, desolvation temperature was 250 °C, cone gas flow was 50 L/ h, and desolvation gas flow was 550 L/ h. MS/MS scan mode was used in instrument to obtain the optimized selectivity and sensitivity. The characteristic ion of SAG is *m/z* 299.0770. The quantitative analyses of SAG in tea leaves were based on the curve of SAG standard and the calibrating of peak area was based on the Leucine-Enkephalin.

4. Determination of Subcellular Distribution of SA and SAG

The tea leaves from *C. sinensis* cv. Jinxuan plucked in March 2019 were fractionated using a nonaqueous procedure according to the published studies with a slightly modified [3–6]. The finely powered tea leaves (4 g, fresh weight) was placed into the lyophilizer at 0.02 bar and -50 °C for 3 days. The dry powder was resuspended in tetrachlorethylene-heptane mixture (20 mL, 66:34 (v/v); density = 1.3 g/cm^3; the solvents were stored with 3 Å molecular sieve) and ultrasonicated for 2 min with 6 cycles of 10 s pules and 10 s breaks at 65% power. The suspension was filtered through nylon net with a pore size at 20 μm, washed the net 3 times with 10 mL of heptane, and centrifuged for 10 min at 3,200 *g* and 4 °C. After centrifugation, the organic supernatant was removed and the pellet was resuspended in C_2Cl_4/C_7H_{16} mixture (3 mL, 66:34 (v/v)). Aliquots (500 μL with 10×50 μL) were withdrawn for determination of metabolites and enzyme activity in the unfractionated material, and the remaining 2.5 mL of the suspension was loaded on the top of the gradient. A linear gradient (25 mL, for leaf tissue between 1.43 and 1.50 g/cm^3) was made using a gradient former connected to a peristaltic pump. The gradients were centrifuged for 1 h at 3,800 *g* at 4 °C. The fractions (F1 to F5, 4-6 mL for each fraction) was carefully removed from the top using Pasteur pipettes into a clean 50 mL tube. Three volumes of C_7H_{16} were added to each tube and the mixture was mixed well. The suspensions were centrifuged for 10 min at 3,200 *g* at 4 °C in a swing-out-rotor centrifuge. The supernatants were discarded and the pellet was resuspended in 5 mL C_7H_{16}, and 10 aliquots of 500 μL of the suspension were transferred into 2 mL tubes. The sample in tube was dried by N_2 for 1 hour, and then extracted for assay of metabolites (SA and SAG) and enzymes (proteins).

The metabolites (SA and SAG) from the dried samples were resolved in 1 mL ethyl acetate, and subjected to ultrasonic treatment for 20 min. After centrifugation, 800 μL su-

pernatant was collected and dried using under a stream of nitrogen. The residue was resolved in 100 μL methanol. The solutions were filtered through a 0.22 μm membrane, and analyzed by UHPLC (Ultimate 3000, Thermo Scientific, Waltham, MA, USA) connected with a triple quadrupole mass spectrometer (TSQ Endura, Thermo Scientific, Waltham, U.S.A.). The sample (2 μL) was injected and separated on a Hypersil GOLD C18 column (2.1 mm × 100 mm i.d., 1.9 μm). The flow rate was 0.3 mL/min. The column temperature was 40 °C. The mobile phase contained acetonitrile solution (A) and water with 0.2% (*v*/*v*) formic acid (B) with an initial condition of 95% of mobile phase B. The linear gradient elution was carried out as follows: 0–3 min, 95% B; 3–20 min, 95%–5% B; 20–23 min, 5% B; 23–23.1 min, 5%–95% B; 23.1–26 min, 95% B. The electrospray ionization (ESI) was operated in the negative ion mode, and the optimal conditions were peak width resolution set at 0.7 *m*/*z*, spray voltage set at 2500 V, sheath gas pressure set at 35 units, auxiliary gas pressure set at 15 units, and vaporizer temperature set at 300 °C. Selected reaction monitoring (SRM) mode was used in MS/MS instrument to obtain the optimized selectivity and sensitivity. The SRM transitions of SA and SAG are listed in Table 1. The quantitative analyses of SA and SAG were based on calibration curves obtained from the authentic standards.

Proteins from the dried samples were extracted by buffer A (50 mM Hepes-NaOH pH 7.4; 1.5 mM PMSF; 1 g/L PVPP; 1 mM EGTA; 1 mM EDTA; 2 mM aminocaproic acid; 2 mM benzamidine; 5 mM $MgCl_2$; 0.1 % Triton X-100; 10 % glycerol) for determined the contents of proteins of GAPDH (plastid marker), UGPase (cytoplasm marker), and Cytochrome C oxidase (mitochondria marker); Proteins from the dried samples were extracted by buffer B (0.5 M sodium acetate pH 5.0 adjusted using glacial acid) for determined the contents of proteins of AP (vacuole marker).

References

[1] Zeng, L.; Wang, X.; Liao, Y.; Gu, D.; Dong, F.; Yang, Z.Formation of and changes in phytohormone levels in response to stress during the manufacturing process of oolong tea (*Camellia sinensis*). *Postharvest Biol. Tec.* **2019**, *157*, 110974.

[2] Zeng, L.T.; Zhou, Y.; Gui, J.D.; Fu, X.M.; Mei, X.; Zhen, Y.P.; Ye, T.X.; Du, B.; Dong, F.; Watanabe, N.; Yang, Z. Y. Formation of volatile tea constituent indole during the oolong tea manufacturing process. *J. Agr. Food Chem.* **2016**, *64*, 5011–5019.

[3] Zhou, X.C.; Zeng, L.T.; Chen, Y.J.; Wang, X.W.; Liao, Y.Y.; Xiao, Y.Y.; Fu, X.M.; Yang, Z.Y. Metabolism of gallic acid and its distributions in tea (*Camellia sinensis*) plants at the tissue and subcellular levels. *Int. J. Mol. Sci.* **2020**, *21(16)*, 5684.

[4] Farré, E.M.; Tiessen, A.; Roessner, U.; Geigenberger, P.; Trethewey, R.N.; Willmitzer, L. Analysis of the compartmentation of glycolytic intermediates, nucleotides, sugars, organic acids, amino acids, and sugar alcohols in potato tubers using a nonaqueous fractionation method. *Plant Physiol.* **2001**, *127*, 685–700.

[5] Krueger, S.; Steinhauser, D.; Lisec, J.; Giavalisco, P. Analysis of subcellular metabolite distributions within *Arabidopsis thaliana* leaf tissue: a primer for subcellular metabolomics. In *Arabidopsis Protocols*; Sanchez-Serrano, J.J., Salinas, J., Eds.; Humana Press: Totowa, NJ, USA, **2014**; pp. 575–596.

[6] Stitt, M.; Lilley, R.M.; Gerhardt, R.; Heldt, H.W. Metabolite levels in specific cells and subcellular compartments of plant leaves. In *Methods in Enzymology*; Academic Press, New York, USA: **1989**; Volume 174, pp. 518–552.
